# Socioeconomic factors associated with severe acute malnutrition in Jamaica

**DOI:** 10.1371/journal.pone.0173101

**Published:** 2017-03-14

**Authors:** Debbie S. Thompson, Novie Younger-Coleman, Parris Lyew-Ayee, Lisa-Gaye Greene, Michael S. Boyne, Terrence E. Forrester

**Affiliations:** 1 Tropical Medicine Research Institute, The University of the West Indies, Mona, Jamaica; 2 Mona GeoInformatics Institute, The University of the West Indies, Mona, Jamaica; 3 UWI Solutions for Developing Countries, The University of the West Indies, Mona, Jamaica; Hospital Universitario de la Princesa, SPAIN

## Abstract

**Objectives:**

Severe acute malnutrition (SAM) is an important risk factor for illness and death globally, contributing to more than half of deaths in children worldwide. We hypothesized that SAM is positively correlated to poverty, low educational attainment, major crime and higher mean soil concentrations of lead, cadmium and arsenic.

**Methods:**

We reviewed admission records of infants admitted with a diagnosis of SAM over 14 years (2000–2013) in Jamaica. Poverty index, educational attainment, major crime and environmental heavy metal exposure were represented in a Geographic Information System (GIS). Cases of SAM were grouped by community and the number of cases per community/year correlated to socioeconomic variables and geochemistry data for the relevant year.

**Results:**

375 cases of SAM were mapped across 204 urban and rural communities in Jamaica. The mean age at admission was 9 months (range 1–45 months) and 57% were male. SAM had a positive correlation with major crime (r = 0.53; P < 0.001), but not with educational attainment or the poverty index. For every one unit increase in the number of crimes reported, the rate of occurrence of SAM cases increased by 1.01% [Incidence rate ratio (IRR) = 1.01 (95% CI = 1.006–1.014); P P<0.001]. The geochemistry data yielded no correlation between levels of heavy metals and the prevalence of malnutrition.

**Conclusion:**

Major crime has an independent positive association with severe acute malnutrition in Jamaican infants. This could suggest that SAM and major crime might have similar sociological origins or that criminality at the community level may be indicative of reduced income opportunities with the attendant increase in poor nutrition in the home.

## Introduction

Severe acute malnutrition (SAM) is defined by a very low weight for height (below -3 z scores of the median WHO growth standards), by visible severe wasting, or by the presence of nutritional oedema [1]. SAM is globally the most important risk factor for illness and death [2]. In 2014, the World Health Organization reported that 45% of all deaths in children under the age of 5 years are linked to malnutrition [3]. SAM is observed most frequently in developing countries but has been described with increasing frequency in hospitalized and chronically ill children in the United States [4]. While not an indicator of SAM, the prevalence of wasting (weight for height more than two standard deviations below the median for the international reference population) in children under age 5 years in Jamaica was 2.50% as of 2012. Its highest value over the past 34 years was 8.90% in 1993, while its lowest value was 1.80% in 2007 [5].

Socioeconomic factors such as poverty, education, gender inequality and access to water and sanitation, are important determinants of health outcomes in many low-income countries. The scientific literature is replete with evidence of associations between malnutrition, poverty and low maternal education [6–8]. Persons of lower socioeconomic status are most vulnerable to food insecurity since purchasing power is a main determinant of the ability to afford nutritious food. In 2012, the World Bank reported that 19.9% of the Jamaican population lives below the poverty line; an increase from 9.9% in 2007 [9]. The result is that inequality has risen, in many instances heightening vulnerabilities of the most-at-risk populations, including women and young people [10].

There are several reports of an association between childhood malnutrition and prolonged conflict [11,12] due to forced displacement and decreased food security. This association has been shown to be independent of parents' educational level or employment status [13]. While not in a state of prolonged conflict, some areas of Jamaica experience high levels of crime and violence [14]. Jamaica has had one of the highest murder rates in the world for many years, according to United Nations estimates [15]. However, major crimes during the years 2000–2012 have been reported as steadily declining [16]. In 2000, major crimes in Jamaica stood at 16,469 and by 2012 it fell by 35.1 percentage points [16]. Based on the statistics, on average, 1,372 major crimes were committed on a monthly basis in 2000 and this fell to 891 on a monthly basis in 2012 [16]. We are unaware of any reported association between the prevalence of SAM and high levels of major criminal activity (i.e. murder, rape, shooting and robbery).

Heavy metals are able to cross the placenta and accumulate in fetal tissues [17]. This exposure to several heavy metals during pregnancy has been shown to be harmful to the developing fetus, for example, lower birth weight is a documented adverse effect of lead exposure during pregnancy [18]. Similarly, gestational cadmium exposure has been associated with low birth weight (2500g) [19] as was moderate arsenic exposures from drinking water (<50 micro g/L) during pregnancy [20]. Postnatal exposure can also occur via breast milk and ingestion of leaded paint, soil contaminated with lead and water carried in lead pipes. Early life exposure to toxic heavy metals such as mercury, lead, arsenic and cadmium, has been documented to cause neurological and psychological effects in children. Additionally, lead and other heavy metals are absorbed more readily in the presence of malnutrition [21]. The combination of environmental exposures to heavy metals and SAM is recognized as a potential contributor to the compromised neuronal development with consequent deficit in cognitive function in children with SAM. Previous studies have reported a high level of several trace elements and heavy metals in the soil of Jamaica [22] including lead [22], arsenic [22] and cadmium [23]. However, there is little data linking the co-occurrence of heavy metals and the prevalence of SAM in Jamaica.

We hypothesized that the occurrence of severe acute malnutrition is more common in communities with lower socio-economic status and higher levels of major crime. We also hypothesized that SAM co-exists in areas with higher mean soil concentrations of lead, cadmium and arsenic. We therefore examined the geographic distribution of cases of SAM, as well as the relationships (at the community level) between number of cases of SAM and socio-economic variables (poverty index, educational attainment), major crime and heavy metal concentration in soil using geo-mapping.

## Materials and methods

### Study design/measurements

The Tropical Metabolism Research Unit (TMRU) in Kingston, Jamaica has been treating children with SAM in a metabolic ward since 1956. The TMRU is the only specialist, in-patient, nutritional rehabilitation centre in Jamaica and receives referrals from the entire island including hospitals and primary health care centres. The protocol for the rehabilitation of SAM has been in use since the 1970s and utilizes three phases of treatment; maintenance, rapid catch up growth and acclimatization to an age appropriate diet. For this study we examined the ward records of children admitted over a 14 year period (2000–2013). The study protocol was approved by the Faculty of Medical Sciences/ University Hospital of the West Indies Ethics Committee. Written informed consent was provided by next of kin on admission and patient records were anonymized prior to analysis.

Malnutrition was diagnosed using the Wellcome Criteria [24] i.e. children presenting with a weight-for-age of 60–80% and oedema are diagnosed with kwashiorkor, while those with marasmus have a weight-for-age < 60% without oedema. Marasmic-kwashiorkor children have a weight-for-age < 60% and oedema. Patients were admitted if they met the diagnostic criteria for any of the clinical forms of SAM (i.e. undernourished, marasmus, kwashiorkor, marasmic-kwashiorkor or edematous malnutrition) with no underlying medical cause (i.e. primary malnutrition). Data relating to age and address at admission, sex and clinical diagnosis were taken from admission records. Addresses were then tallied by communities in the Mona GeoInformatics Institute (MGI) of the University of the West Indies database to provide a spatial dimension to statistics and general contextual data on SAM patients. Data relating to community boundaries, population and poverty were obtained from the Planning Institute of Jamaica (PIOJ, 2002). The database that was utilised for this study did not contain the community populations in absolute terms. Poverty was defined as the percentage of persons in a community living below the poverty line. The 2001 Jamaica Survey of Living Conditions was used to identify several variables that could be used to estimate poverty. These variables were then used to ascribe consumption levels for each individual in the 2001 Population Census. The prevalence of poverty for the communities was subsequently calculated [25]. Data relating to educational attainment was obtained from the Statistical Institute of Jamaica (STATIN) and was derived from both the 2001 and 2011 Population Census. Educational attainment for the community was defined as the percentage of persons in a community who had achieved tertiary level education in the respective years. Crime data was obtained from the Jamaica Constabulary Force (JCF) and was defined as the number of reported cases of murder and shootings in a community in a given year. This data is captured annually, with data for the parishes of Kingston and St. Andrew reported from 2000 to present and island-wide crime data reported from 2007 to present. No crime data was available for the year 2006 for the entire island. Geochemistry data in the form of levels of heavy metals in the soil was obtained from the Geochemical Atlas of Jamaica published by International Centre for Environmental and Nuclear Sciences at the University of the West Indies [26].

### Data management and analysis

The primary outcome variable for these analyses was the number of cases of SAM from each community in each year of the period under study (2000–2013), admitted to the TMRU ward. The total number of cases of SAM in each community for the entire was obtained and used as the outcome variable for initial data analysis. As there was no crime data for 2006 and from rural communities before 2007, cases for these periods were excluded from the calculation of this total prior to use in subsequent analyses that used crime data. The independent variables used to explain variation in the SAM data were percentage of persons in a community living below the poverty line as estimated from the 2001 Jamaica Survey of Living Conditions; percentage of persons in a community who had achieved tertiary level education in 2001; the respective soil levels of lead, arsenic and cadmium (in parts per million) as measured in the years 1987 and 1988 and the total number of reported crimes for each community for the entire period under study. Thus, in the data set used for one set of analyses, there was a single observation for each community. Communities were categorised according to whether they provided crime data prior to 2007. Values of the outcome and all independent variables were placed in a common geographic information system, after which various analyses were performed. These included simple overlays and extractions, as well as queries and filter operations. Summary statistics for all variables were determined. Spearman rank correlation coefficient was estimated to quantify the strength of the association between the number of cases of SAM in each community over the period and each of the explanatory variables. For each of the variables that had a statistically significant correlation with the number of cases, Poisson regression models were used to further examine the association of these variables with the occurrence of SAM in the communities. Model creation accounted for the effect of clustering due to availability of crime data prior to 2007. Thus, the robust estimates of standard errors allowed for intragroup correlation. Forward stepwise estimation was used to determine selection of the final model.

Subsequent analyses attempted to account for the effects of clustering due to community and of time on variation in the outcome. The data provided were from communities across the island of Jamaica. All parishes were represented in the sample. The number of SAM cases from each community admitted to the TMRU ward in each year, number of crimes reported for the respective year, proportion of community residents below the poverty line for that year, and the proportion of the community with tertiary level education in 2001 were the variables examined in these next analyses. Yearly data were available for 2000 to 2005 and 2007 to 2013 for the parishes of Kingston and St. Andrew (KSA) and for 2007 to 2013 for other parishes (OP). Thus, subgroups analyses were carried out using data from KSA and from OP.

The distribution of the number of SAM cases disaggregated over community and time was examined, and values for Akaike Information Criterion (AIC) as well as deviance were used to compare models to determine the regression method that would most appropriately model the association between the outcome and the explanatory variables of interest. Summary statistics estimated included within and between cluster (community) estimates of variance. Poisson regression models with coefficient of the natural logarithm of the time variable constrained to 1 and variance estimates adjusted for intra-cluster correlation, were used to examine the nature of the relationship between number of SAM cases and the explanatory variables. Initially, each model had a single explanatory variable. Where the explanatory variables had p-values less than 0.3, estimates of their effects on the outcome variable were adjusted for year of admission to the ward.

It is understood that, as the measures were collected over a number of years, any statistically significant correlation identified via the Spearman rank correlation analysis indicated earlier, could be the result of the autocorrelation in values of the respective measures. In order to carry out this examination of correlation between measures with any serial correlation removed, the variables that were gathered over time were collapsed across communities to yield a single value for each year, a time series of measurements. We estimated the Pearson correlation coefficients for association between the first differenced values of the time series of the outcome and explanatory variables and autocorrelation coefficients for the respective time series. Statistical significance of the latter was determined using the portmanteau (Q) test. We also examined the association between the outcome and the explanatory variables using regression models of the first differenced time series of values.

Stata versions 12 and 14 (College Station, TX: StataCorp LP) were used to analyze the data.

## Results

**Fig 1:**
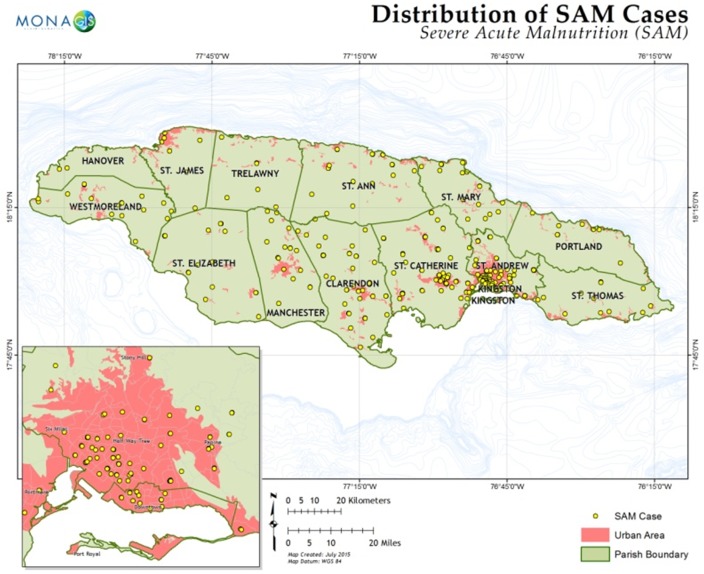
Distribution of cases of severe acute malnutrition.

408 children were admitted with a diagnosis of SAM during the period under review. Addresses of a total of 375 out of the initial 408 patients were mapped. Those excluded represented errors in patient address (i.e. missing or vague address entries) which prevented 100% mapping. Cases of malnutrition were admitted from all 14 parishes in Jamaica. The 375 cases were distributed across 204 out of 829 urban and rural communities in Jamaica (Fig 1). Rural communities, as defined by the 1998 Land Use Map of Jamaica by the Forestry Department, accounted for a total of 149 patients and urban communities accounted for 226 patients. After exclusion of participants of data from communities and years for which there was no corresponding crime data, 153 communities provided data for regression analyses of associations between variables and time series analyses. The number of cases of SAM in a given community in a given year ranged from 1 to 3 while the number of cases in a community over the period 2000 to 2013 ranged from 1 to 9 with a median value of 1. Table 1 gives summary statistics and autocorrelation coefficient (ac) for the distribution of the number of SAM cases within and across parish subgroups. Over the years, number of cases range form 1–2 for KSA and 1–3 for OP. Estimates of the overall and between-group variance as can be estimated from the standard deviation values presented, are all lower than the mean values shown suggesting that there may be under-dispersion in the distribution and data possibly follow a quasi-Poisson rather than a Poisson distribution. There is strong (ac = 0.66) and statistically significant (*P* < 0.01) serial correlation in the distribution of number of SAM cases over time but this is driven by the serial correlation in the measurements from KSA. (Table 1)

**Table 1:** Summary statistics and autocorrelation coefficient for the number of cases of severe acute malnutrition as assessed in 153 Jamaican communities.

Index	No. Communities	No. observations	Mean± SD	Range	Autocorrelation Coefficient (Lag 1)
All Parishes	153	214	1.12±0.34(o)	1–3	0.66**
			1.12±0.30(b)	1–3	
KSA	47	85	1.14±0.35(o)	1–2	0.54*
			1.14±0.28(b)	1–2	
OP	106	129	1.11±0.34(o)	1–3	0.12
			1.11±0.31(b)	1–3	

KSA = Kingston and St. Andrew parishes, OP = Other parishes. (o)–overall, (b)–between communities;

* = *P* < 0.05,

** = *P* < 0.01,

*** = *P* < 0.001

Of the 375 patients mapped, the mean age at admission was 9 months (range 1–45 months) and 57% were male. 12 deaths were documented over the period yielding a mortality rate of 3.2%. Table 2 combines the summary statistics of the socio-economic and geochemistry variables and the correlation coefficients of those variables and the prevalence of SAM by community.

**Table 2:** Summary statistics and Spearman rank correlation coefficient for the associations between socioeconomic and geochemical variables and number of cases of severe acute malnutrition as assessed in up to 204 Jamaican communities.

Index	Number of Communities	Mean	SD	Correlation Coefficient
Age (months)	204	9.1	4.8	0.12
Poverty Index	204	24.2	11.8	-0.13
Tertiary education (2001)	204	7.0	7.2	0.10
Tertiary education (2011)	204	7.4	6.6	0.01
Crime^+^	153	12	24.1	0.53***
Lead (ppm)	195	33.64	34.13	0.001
Arsenic (ppm)	195	16.9	21.7	0.002
Cadmium (ppm)	195	13.1	31.7	0.002

*** *P* < 0.001,

^+^Median (IQR) = 3 (1, 12)

**Fig 2:**
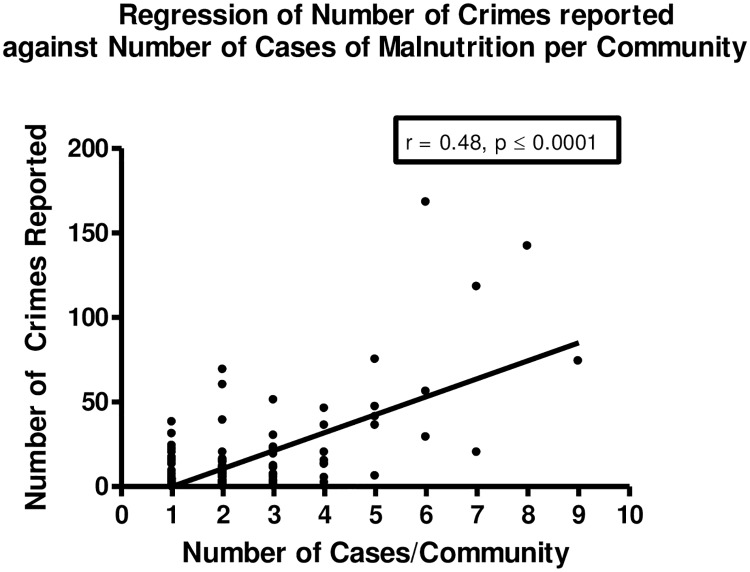
Scatter plot of number of cases from each community against number of crimes reported.

The number of SAM cases had a positive correlation with level of major crime (r = 0.53; *P* < 0.001), (Table 2, Fig 2), but not with educational attainment or the poverty index. There was also a positive association between level of crime and number of persons with tertiary level education in 2001 (Spearman’s rho = 0.26, *P* = 0.0013) while there was a negative association between levels of crime and proportion of persons below the poverty line (Spearman’s rho = -0.32, *P <* = 0.0001).

As estimates of these correlation coefficients could be driven by the serial correlation in the measurements obtained over time, autocorrelation analysis was carried out. Also, the correlation between the measures in the absence of the autocorrelation was also estimated. For the 153 communities used in the second phase of the analyses, autocorrelation coefficients with lag of 1 was estimated for the crime and poverty measures and the correlation of these measures, when first differenced, with number of SAM cases, also first differenced, is shown in Table 3, as is their correlation with proportion of community residents with tertiary level education in 2001. There was no evidence of statistically significant serial correlation in the crime and poverty measures. The association between crime and number of cases of SAM was retained even after removal of the serial correlation within the time series with a coefficient of 0.62 for the entire sample of communities but driven by the strong, statistically significant correlation (ρ = -0.71) in KSA. Neither the association between crime and tertiary education nor poverty was retained when the serial correlation was removed. There was a statistically significant negative correlation between tertiary education share and poverty index after the latter was first differenced. (Table 3)

**Table 3:** Autocorrelation coefficient (lag 1) for the poverty and crime indices and Pearson correlation coefficient for correlation of these variables with the number of cases of severe acute malnutrition as assessed in up to 153 Jamaican communities.

Index	Autocorrelation Coefficient (Lag 1)	Correlation with No. SAM Cases (first differenced)	Correlation with Tertiary Education share(2001)
All Parishes Crime	0.33	0.62*	-0.07
Poverty Index	-0.33	0.60^+^	-0.33
Kingston & St. Andrew Crime	0.30	0.71*	0.06
Poverty Index	0.06	-0.13	-0.72*
Other Parishes Crime	0.14	0.60	-0.48
Poverty Index	-0.21	0.76^+^	-0.13

* = *P* < 0.05,

** = *P* < 0.01,

*** = *P* < 0.001,

^+^ = *P* < 0.1

For KSA, the first and second difference estimators for all variables measured over time—number of cases of SAM admitted to the ward, the poverty index, number of crimes reported, and percentage of persons who attained tertiary level education in the respective years were estimated using a null regression model. Though not statistically significant, first difference estimators suggested that number of cases of SAM admitted was declining at about one per year and the number of crimes reported was falling at about 5 per year. Poverty was also declining at rate of less than one percentage point per year, but the measures of education were increasing. Second difference estimators for number of cases, number of crimes, and poverty indicated that the respective rates of decline were occurring at a decreasing rate that was not significantly different from 0.

Having removed the effect of time on the measures, regression analysis was carried out to determine whether the relationship between number of SAM cases was associated with any of the variables. Simple linear regression of the first differenced values of number of SAM cases as the outcome variable against the first differenced values of each of the poverty index, number of crimes reported, percentage of persons who attained tertiary level education in the respective years was carried out. The model with first differenced values of crime yielded the lowest AIC (= 55.6) of all the models and showed a statistically significant association between SAM and crime. Unadjusted estimates showed that each one unit increment in change in number of crimes was associated with a 0.06 (0.016 to 0.106; *P* = 0.014) unit change in the decline in the SAM cases. In other words, the rate of decline in number of SAM cases increases as rate of decline in number of crimes increases. This association was retained with β = 0.075 (0.029 to 0.12; *P* = 0.006) even after adjusting for poverty level and tertiary education share in 2001, both first differenced. The model yielding these results had with lowest AIC (= 54.6) when compared with all other possible models.

None of the associations obtained for KSA using regression models of first differenced values, were obtained for the OP.

For the two phases of the analyses, the stepwise estimation procedure (probability of entry to model = 0.3), the Deviance and Pearson goodness-of-fit tests, and the Akaike Information Criterion (AIC) were used to determine the best final Poisson regression model which also produced robust estimates of standard errors. The Poisson regression model was deemed the appropriate method for regression analysis of these count data as a negative binomial model revealed that the over-dispersion parameter was not significantly different from zero, the value to which this value is constrained for the Poisson regression model.

**Fig 3:**
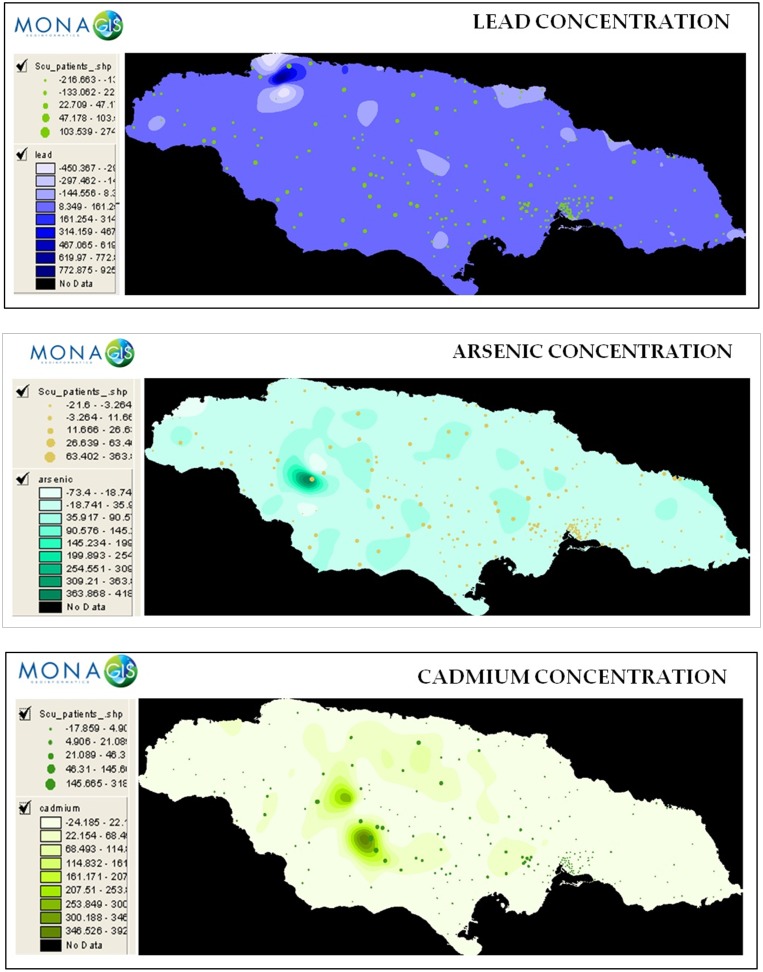
Concentration of lead, arsenic and cadmium in communities across Jamaica (parts per million).

For phase 1 models in which neither the effect of clustering due to community nor time were accounted for, each explanatory variable was placed in a model as the single predictor of number of SAM cases. Table 4 gives the unadjusted incidence rate ratios and AIC values for models having the crime variable alone or with other explanatory variables in the regression model. The poverty index and number of crimes for the period under study were the variables in the final model which had the lower AIC of the two models presented and showed that the rate of occurrence of malnourished cases was higher in communities with higher levels of crime. For every one unit increase in the number of crimes reported, the rate of occurrence of SAM cases increased by 1% [Incidence rate ratio (IRR) = 1.01 (95% CI = 1.006–1.014); *P* < 0.001]. This association remained even after adjustment for poverty index. Geochemistry data was available for a total of 195 of the 204 communities (Fig 3). The geochemistry data yielded no correlation between levels of heavy metals and the number of cases of malnutrition (Table 2). Therefore, the geochemistry variables were not used in the regression models.

**Table 4:** Unadjusted incidence rate ratios with 95% confidence intervals and Akaike Information Criterion, from Poisson regression models, for the association of explanatory variables with rate of admission to the TMRU ward from 153 communities across Jamaica (2000–2013).

Explanatory Variable	Unadjusted IRR (95% CI)	AIC (after adding to model with crime)
Crime	1.01(1.006–1.013)***	410.02^@^
Poverty Index	1.00(0.99–1.01)	411.25
Tertiary education share (2001)	0.998(0.98–1.02)	412.00

* = *P* < 0.05,

** = *P* < 0.01,

*** = *P* < 0.001,

^+^ = *P* < 0.1;

^@^: AIC for crime as only explanatory variable.

Thus far, results of regression models have accounted for neither of the effect of clustering on the variance estimates nor the effect time on the rate of admission of SAM cases. Subsequent analyses sought to fill these gaps.

Attempts to jointly model the longitudinal and clustered nature of the data using the generalized estimating equation for longitudinal data (based on either of the negative binomial or Poisson distribution with the log link), while incorporating the autoregressive correlation structure, also failed to converge. This is likely due to the imbalance in the number of years for which communities had SAM cases on the TMRU ward. Only 10 of the 153 communities had more than two observations over the 14-year period with only one providing number of SAM cases for a maximum of 7 years. Use of the negative binomial regression models with variance estimation adjusted to account for clustering in communities revealed that the dispersion parameter was not significantly different from zero and deviance statistics showed that the negative binomial model was not a better fit than obtained by use of the Poisson regression model. Thus, analysis proceeded using the Poisson regression model.

Bivariate analysis examined, initially, whether each of tertiary education share in each community in a given year, percent below poverty line, number of crimes reported, were significantly associated with number of SAM cases were admitted to the wards from the communities. With year of admission as the exposure or offset variable for the Poisson models, analysis revealed association between crime and number of cases admitted approaching significance for KSA only. In KSA, at the 10% significance level, every one-unit increment in the number of crimes reported can be expected to increase the number of cases admitted by 1% (IRR = 1.01, 95% CI = 0.999–1.015, *P* = 0.093) (Table 5) and significance at this level was retained even after year of admission was added to the model. There was no significant association between number of SAM cases and any of the other explanatory variables within or across parish subgroups. The final models for these data had number of crimes and the year of admission as explanatory variables. For KSA, compared with the reference year 2000, for the year 2008 only the rates of admission were significantly different (IRR = 1.49, 95% CI = 1.05–2.11; *P* = 0.026). In the other parishes, the rates tended to be lower for the years following the reference year 2007, and the differences achieved statistically significance for 2013 only different (IRR = 0.86, 95% CI = 0.75–0.99; *P* = 0.038).

**Table 5:** Unadjusted incidence rate ratios with 95% confidence intervals, from Poisson regression models with variance correct for intragroup correlation, for the association of explanatory variables with rate of admission to the TMRU ward from 153 communities across Jamaica (2000–2013).

Explanatory Variable	All Parishes	KSA	OP
Crime	1.001(0.999–1.004)	1.007(0.999–1.015)^+^	1.00(0.998–1.002)
Poverty Index	1.001(0.998–1.004)	1.002(0.997–1.007)	1.00(0.997–1.004)
Tertiary education share (2001)	0.999(0.993–1.005)	0.999(0.992–1.006)	0.997(0.988–1.004)

* = *P* < 0.05,

** = *P* < 0.01,

*** = *P* < 0.001,

^+^ = *P* < 0.1

## Discussion

In this retrospective study, using methods that did and did not correct for possible effects of time and clustering due to community, we demonstrated a positive relationship between major crime and the number of cases of severe acute malnutrition in communities across Jamaica. Communities with higher levels of crime can therefore be expected to have higher rates of SAM. There are several reports of associations between early childhood malnutrition and subsequent low IQ and anti-social behavior [27]. This may be due to persistent neurocognitive effects of malnutrition. There are also many reported associations between childhood malnutrition and prolonged conflict [11,12]. Additionally, mothers' report of domestic violence was associated with an increased prevalence of chronic malnutrition in children under five years old in Peru [28] and similar findings were reported in India [29]. However, the authors are unaware of any other studies in the literature reporting crime as an independent correlate of severe acute malnutrition.

One possible explanation for this association could be that SAM and major crime might have similar socio-economic origins. It is conceivable, for example, that childhood economic disadvantage could be a determinant of crime as well as SAM [30]. Additionally, criminality may reduce income opportunities in communities, thereby limiting a family's capabilities to provide adequate childhood nutrition. While we were able to show an association between levels of crime and cases of SAM within communities, this observational study cannot establish causality. However, Harriott (2001) described the concentration of urban joblessness, poverty, poor educational opportunities and generalized marginalization in Jamaica as being criminogenic [31]. We posit, therefore, that these might represent modifiable sociocultural factors that can be used as the basis for interventions aimed at curbing the problem of malnutrition.

Socio-economic factors such as poverty and low educational attainment were not correlated with the occurrence of SAM in our study. We believe there are several explanations for this unexpected outcome. Poverty index was derived at a community level and not an individual household level. Additionally, the use of community poverty indices and not individual/household consumption patterns or income in calculating poverty index might have contributed to these findings. Similarly, the fact that low educational attainment was not correlated to SAM may again be explained by the use of community data, where data relating to maternal education might have been more relevant. This suggests that family/household level data relating to education and poverty might be a better predictor of the outcome of interest. Additionally, we may have been underpowered to detect these associations.

Our data yielded no correlation between levels of heavy metals and the number of cases of malnutrition. At a mean admission age of 9 months, the most likely means of environmental heavy metal exposure in our study population would be trans-placental or breast milk transfer. While we do not have data relating to breastfeeding in our study population, the prevalence of exclusive breastfeeding (at least 6 months) was 22.2% among mothers in rural Jamaica [32] and 29.9% in mothers attending an urban hospital clinic [33]. Kumar et al demonstrated that delayed initiation of breast-feeding and improper weaning are significant risk factors for under nutrition among children under the age of 5 years [34]. It is possible that these improper breastfeeding and weaning practices could explain the lack of association between malnutrition and heavy metal concentrations even in communities with high mean concentrations.

Our study had several limitations. First, our analyses were restricted to children who were admitted to our hospital while the data relating to poverty, education and crime were for the general population. Even though our hospital is the main referral centre in the island, we acknowledge a potential selection bias, thus our sample may not be representative of the entire population of children with SAM with results indicating a decline in the relative numbers of children from parishes outside of KSA, over time. Additionally, the study could have been strengthened by the collection of data relating to parental educational attainment and household income at the time of admission as opposed to the use of community data. Community population data would also have been helpful in the analysis. Additionally, data relating to levels of lead, arsenic and cadmium were from 1987–1988. Nevertheless, we have demonstrated an independent association between major crime and severe acute malnutrition in Jamaican children. While crime at the community level is associated with severe acute malnutrition it is likely that household factors are also associated. In addition, the finding of the relationship between crime levels in communities and numbers of SAM cases has been further bolstered by analyses that accounted for the effect of clustering due to community and removal of serial correlation in the distribution of the respective variables.

Our findings have added to the discourse relating to the socioeconomic associations of severe acute malnutrition by identifying communities that may be more vulnerable. Our data suggest the need for community intervention strategies but intervention at the level of the household might also be critical. A thorough understanding of the contributors to childhood malnutrition will assist policy-makers in designing effective strategies which will in turn trigger better gains in child health and a further reduction in child mortality.

All authors revised the manuscript for intellectual content and approved the final version.

## Supporting information

S1 File DATASET_tmru_patients_final_2015_07_12_2_collapsed by Community only. (XLS)

S2 File Cases by time all parishes_2016_10_27_1. (XLS)

S3 File Cases by time_ksa vs rest_2016_10_25_1. (XLS)

S4 File DThompson et al_SEF and SAM_Community. (XLS)

S5 File DThompson et al_SEF and SAM_Community_year. (XLS)

S6 File Socioeconomic factors and SAM. (TXT)

S7 File Socioeconomic factors and SAM_sensitivity analyses. (TXT)

S1 Text Licence. (DOCX)

S2 Text License2. (DOCX)
